# Laparoscopic Diaphragmatic Pacing in Spinal Cord Injury Patients with Respiratory Failure: A Saudi Arabian Experience

**DOI:** 10.3390/medsci14030375

**Published:** 2026-07-04

**Authors:** Suha Kaaki, Aya K. Aldayel, Waseem M. Hajjar, Ahmad W. Hajjar, Sami A. Al-Nassar

**Affiliations:** 1 Department of Surgery, Division of Thoracic Surgery, King Saud University, Riyadh 11472, Saudi Arabia; skaaki@ksu.edu.sa (S.K.); whajjar@ksu.edu.sa (W.M.H.); 2 Department of Surgery, Prince Mohammed Bin Abdulaziz Hospital, Ministry of Health, Riyadh 14214, Saudi Arabia; akaldayel@moh.gov.sa; 3 College of Medicine, Alfaisal University, Riyadh 11533, Saudi Arabia; awhajjar@alfaisal.edu

**Keywords:** mechanical ventilation, diaphragmatic pacing, cervical spinal cord injury, ventilator weaning

## Abstract

Background/Objectives: Cervical spinal cord injury (SCI) carries a significant burden in Saudi Arabia, frequently resulting in permanent ventilator dependence and high morbidity. While laparoscopic diaphragmatic pacing (DP) has emerged as an alternative to long-term mechanical ventilation (MV) globally, regional evidence regarding its application within the Middle East remains limited. This study evaluates a single-center cohort of ventilator-dependent cervical SCI patients undergoing laparoscopic DP. Methods: We conducted a retrospective analysis of all ventilator-dependent patients with cervical SCI admitted to a tertiary hospital in Riyadh between 2012 and 2024 who underwent laparoscopic DP after failing traditional weaning attempts. Inclusion criteria required at least 3 months of MV dependence, intraoperative diaphragmatic stimulability and a minimum one-year follow-up post-implantation. Across the entire cohort, the long-term follow-up duration reached a median of 60.0 months (interquartile range [IQR]: 36.0–84.0 months; range: 12.0–120.0 months). Results: Out of 30 initial candidates with cervical SCI, 28 patients (22 males, 6 females; median age 24.0 years (interquartile range [IQR]: 15.0–33.0 years)) were included. Patients had been on MV for a median of 13.0 months (IQR: 10.5–16.0 months) prior to the procedure. Utilizing a combined weaning success rate (complete or partial weaning), 26 patients (92.86%; 95% CI: 77.42–98.01%) were successfully transitioned to the pacing protocol, while 2 patients (7.14%) experienced DP failure. Complete (24 h) daily MV independence was achieved by 18 patients (64.29%), and partial weaning (≥4 h/day of MV-free time) was achieved by 8 patients (28.57%). Age at the time of injury ranged from 5 to 62 years. No major intraoperative or postoperative complications occurred. Minor exit-site skin irritation was observed in 3 patients (10.71%), all of which resolved completely with conservative local care alone without requiring antibiotic therapy. Conclusions: In this selected single-center Saudi cohort of ventilator-dependent cervical SCI patients, laparoscopic DP was feasible and was associated with high rates of partial or complete ventilator-free breathing. Larger prospective multicenter studies with standardized selection criteria, safety reporting, respiratory outcomes, quality-of-life measures, and longer follow-up are needed.

## 1. Introduction

Respiratory failure is one of the major complications of cervical SCI and a frequent cause of associated morbidity and mortality [1]. Depending upon the level of injury, such traumatic cervical lesions can lead to tetraplegia or partial/complete loss of respiratory function [2]. The phrenic nerves originating from the C3, C4, and C5 levels of the spinal cord transmit neural respiratory impulses to stimulate the diaphragm [3]. Hence, traumatic injuries occurring at or above C3 lead to total interruption of communication between the respiratory centers and phrenic nerves, thus causing complete diaphragmatic paralysis. Lesions at the C4/C5 levels mostly result in a partial reduction in diaphragm activity [4,5].

MV via tracheostomy represents the standard of care in cases with complete cervical SCI at or above the C3 level [6], and among patients with complete cervical injuries distal to C3. However, the chronic use of MV in such patients is associated with multiple complications and has raised concerns among the global medical fraternity. Patients on MV have a poor quality of life due to complications like dysphonia [7], olfactory loss, [8] excessive secretions warranting frequent suctioning [8,9], and perpetual noise generated by the ventilator [4,5]. Lower lobes atelectasis, pneumonia, barotrauma, tracheomalacia, and lower life expectancy comprise some of the seriously known adverse effects of MV in cervical SCI patients [4,5].

The DP represents an adjunctive technological option designed to mitigate specific long-term mechanical ventilation restrictions and complications in appropriately selected cervical spine injury populations. It is a device that stimulates the diaphragmatic muscles by generating electrical impulses directed at the phrenic nerves’ endings. This helps to re-establish the lost connection between the phrenic motor neurons and the diaphragm in cervical SCI patients. It represents a more natural method of diaphragmatic stimulation and helps patients achieve respiration in a more physiological manner compared to traditional positive-pressure ventilation [10,11]. Prior clinical literature suggests that the use of DP may support improved survival [12], lower pulmonary complications [5,12], and partial-to-complete independence from MV in selected high-cervical SCI management [11].

Recently, there has been more focus on early DP after SCI with an initial report in 2014 [13]. While a prior pediatric case report from Riyadh described DP in a single 4-year-old child [14], this study represents one of the first Saudi retrospective cohort reports known to the authors to describe a broader institutional series within the Kingdom of Saudi Arabia. Given the regional burden of traumatic SCI resulting from motor vehicle accidents and falls, evaluating localized clinical experience with diaphragm stimulation is essential. This retrospective cohort study describes the feasibility, clinical outcomes, and safety profile of laparoscopic DP placement in a selected cohort of ventilator-dependent cervical SCI patients at a university tertiary care center in Riyadh.

## 2. Materials and Methods

### 2.1. Study Design and Ethical Considerations

A retrospective study was conducted among a cohort of cervical SCI patients, who underwent DP placement at a university tertiary hospital in Saudi Arabia between January 2012 and December 2024. Ethical approval was granted by the Institutional Review Board and Ethics Committee (Protocol: E-24-8673, April 2024), with a waiver of informed consent for retrospective research data use, though explicit clinical consent was obtained for all surgical procedures.

### 2.2. Selection Criteria

The inclusion criteria targeted ventilator-dependent SCI patients (ages 5–62 years) with a minimum of three months of MV dependence and intraoperative diaphragmatic stimulability. Exclusion criteria included a lack of social support, active psychiatric disorders, or significant comorbid medical conditions that precluded surgery.

Operational Definitions of Weaning Outcomes.

Weaning Initiated: Patients who successfully initiated the postoperative DP conditioning protocol and demonstrated an initial reduction in baseline MV dependency.

Partial Weaning: ≥4 h/day of MV-free time; patients typically require nocturnal or intermittent mechanical ventilation support.

Complete/Successful Weaning: Full 24 h daily independence from MV (0 h/day MV requirement) supported continuously by DP.

DP Failure: Complete inability to be weaned off the mechanical ventilator.

Current DP use/Ventilator-free time: In this study, ‘current DP use’ and ‘ventilator-free time’ are used interchangeably to define the subset of weaned patients who achieved independence from mechanical ventilation.

Tracheostomy tubes remained in place throughout the initial inpatient conditioning phase for safety. Weaning was initiated in a monitored hospital setting according to a standardized progressive pacing protocol before transitioning to home-based MV support. Criteria for structural progress evaluation included diaphragm’s strength improvement, stable oxygen saturation (SpO2 ≥ 92%), resting respiratory rates between 12 and 20 breaths per minute, the complete absence of accessory respiratory muscle recruitment, and patient comfort during consecutive trials.

Immediately following surgery, initial device parameters are adjusted by a clinician to find comfortable baseline stimulation levels. Formally trained caregivers then assist the patient in transitioning off the mechanical ventilator for short, designated conditioning sessions during which the NeuRx DPS is activated to progressively exercise the diaphragm and build physical endurance. The total duration of these off-ventilator pacing periods is progressively increased over time based directly on documented improvements in diaphragmatic muscle strength. Because baseline deconditioning and neurological recovery profiles are highly variable, there is no uniform conditioning timeline; the schedule varied transparently by patient, with the specific duration and progression of pacing sessions determined dynamically by each patient’s care team based on individual tolerance and progress. To manage the physiological rise in blood carbon dioxide (CO_2_) levels during conditioning, the mechanical ventilator’s tidal volumes are slowly weaned down in gradual decrements of 25 cc to 50 cc. If initial exhaled tidal volumes are low, the pacing respiratory rate can be increased to manage minute ventilation needs before being adjusted downward as the muscle strengthens. Patient safety is maintained throughout the inpatient and home conditioning phases by continuously tracking oxygen saturation (SpO2 ≥ 92%) via a pulse oximeter, monitoring breathing volumes using a Wright Respirometer, assessing for accessory respiratory muscle recruitment, and ensuring a fully operational backup mechanical ventilator system remains continuously available at the bedside.

### 2.3. Definitive Gateway Selection Criterion

The ultimate determinant for device implantation was intraoperative diaphragmatic stimulability mapping during laparoscopy. If a patient’s diaphragm demonstrated a robust contractile response to diagnostic electrical stimulation, we proceeded with DP implantation; if non-stimulable, the procedure was aborted.

Baseline Characteristics of the Study Cohort (*N* = 28).

The baseline profiles for the evaluated cohort are summarized below (the complete anonymized data for each individual patient can be found in Supplementary Table S1):

Neurological Level of Injury: High-level cervical spine injuries, predominantly localized between C2 and C5, distributed as follows: C2 (*n* = 4, 14.3%), C3 (*n* = 11, 39.3%), C4 (*n* = 11, 39.3%), and C5 (*n* = 2, 7.1%).

Completeness of Injury (ASIA Grade): The vast majority of the cohort presented with ASIA Grade A (complete) injuries (*n* = 23, 82.1%), with a minority classified as ASIA Grade B (incomplete) (*n* = 5, 17.9%). Phrenic Nerve and Diaphragm Integrity Testing: Preoperative lower motor neuron viability was structurally verified in all patients via nerve conduction assessments, electromyography, diaphragm ultrasound, and fluoroscopy prior to surgical confirmation, with 100% of the included cohort demonstrating intact phrenic nerve conduction.

Tracheostomy Status: 100% of the patients had an active tracheostomy tube in place prior to enrollment. All patients in this cohort remained cannulated for life to ensure long-term airway safety and effective secretion management, with routine tracheostomy tube replacements scheduled regularly every 3 to 4 months during long-term follow-up. No patients were decannulated.

Pediatric Governance and Off-Label Compassionate-Use Disclosures:

This cohort includes pediatric patients as young as 5 years old. Because the NeuRx Diaphragm Pacing System is regulatory-cleared primarily for adult populations (aged ≥18 years), all pediatric applications within this series were conducted as off-label, compassionate-use interventions. To ensure strict ethical and clinical oversight, each pediatric case was subjected to a rigorous pre-surgical multidisciplinary governance review. This review panel required a formal consensus among thoracic surgeons, pediatric pulmonologists, pediatric intensive care specialists, and the institutional medical ethics board to verify that the benefits of eliminating positive-pressure ventilation risks outweighed the surgical risks. Legal written informed consent was explicitly obtained from the parents or authorized legal guardians of all minor patients prior to any surgical intervention. Furthermore, for older pediatric patients who possessed the developmental and cognitive capacity to comprehend the nature of the treatment, verbal or written patient assent was systematically obtained in strict compliance with local institutional pediatric bioethics guidelines and Institutional Review Board protocols. While the operational pacing configurations and endurance milestones were individualized based on respiratory tolerance, the intraoperative monitoring parameters, vital sign tracking (including continuous SpO2 monitoring), and bedside backup mechanical ventilation safety protocols for pediatric patients did not differ from those applied to the adult cohort.

The study intended to determine the prevalence of weaning from MV among patients who received a DP implant. Hence, the primary outcome measure of the study was the incidence of weaning from MV after placement of the DP. The other exploratory outcome measures were mean duration of current DP use, impact of age at injury and timing of DP placement on the time required to achieve weaning.

### 2.4. Diaphragmatic Pacemaker

**Figure 1:**
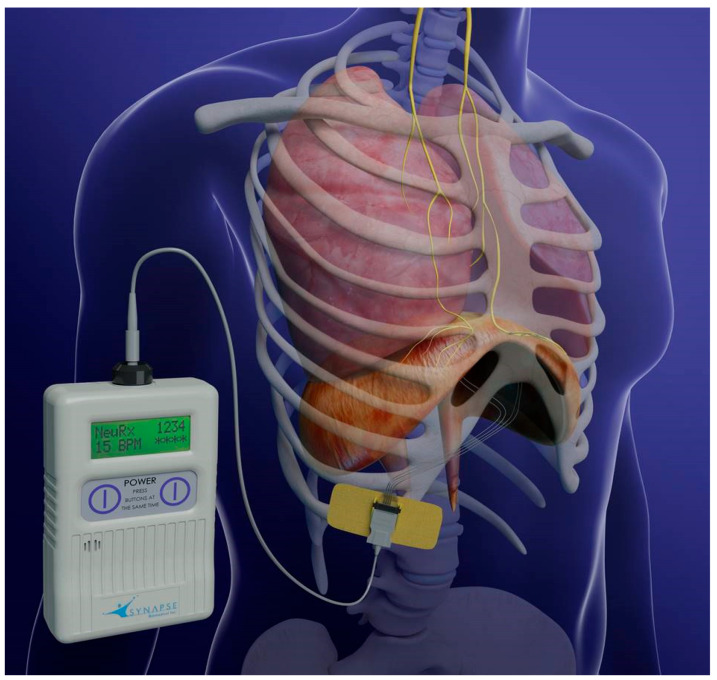
External stimulator of the diaphragm pacemaker. (Synapse Biomedical, Oberlin, OH, USA).

**Figure 2:**
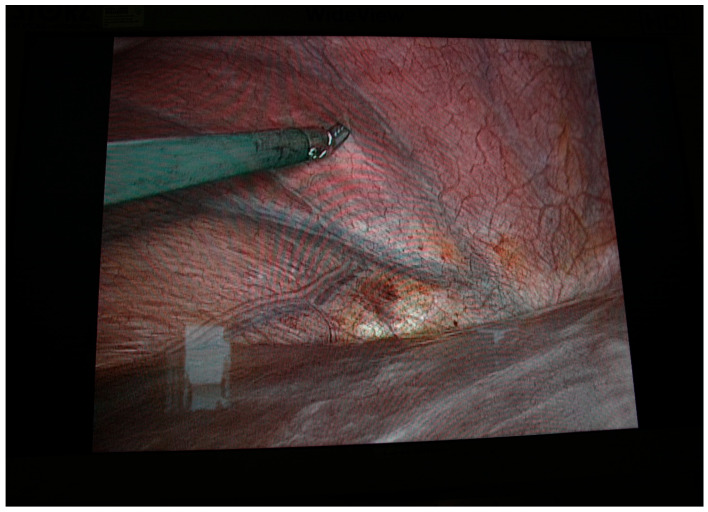
Mapping the motor points of the diaphragm with the maximum contraction. Images reproduced with clinical institutional permission; system properties showcase the NeuRx DPS diagnostic platform.

The NeuRx Diaphragm Pacing System™ (NeuRx DPS™; Synapse Biomedical, Oberlin, OH, USA) was utilized for all patients (Figure 1). Implantation was achieved via standard laparoscopic access, utilizing PermaLoc intramuscular electrodes anchored directly into the bilateral diaphragmatic motor points mapping sites (Figure 2). For a highly detailed, step-by-step description of the surgical procedure, port placements, intraoperative mapping parameters, titrations, external stimulator programming, and device troubleshooting protocols, please refer to “Supplementary File S1”.

Data parameters were collected through a structured, retrospective review of historical electronic medical records and institutional clinical charts. The exact pre-implant duration of mechanical ventilation was verified through historical intensive care unit and respiratory therapy flow sheets. Postoperative metrics—including the duration of active DP usage, complete or partial weaning success categories, and long-term follow-up timelines—were gathered from specialized outpatient follow-up notes. Respiratory dynamics data, specifically the baseline positive-pressure mechanical ventilation tidal volumes and active DP system tidal volumes, were pulled from standardized respiratory therapy charts. Finally, intraoperative parameters and long-term postoperative adverse events (including exit-site skin complications or device mechanical performance) were audited word-for-word from surgical reports and longitudinal clinical follow-up charts.

### 2.5. Outcomes and Statistical Analysis

Continuous variables tracked across the patient registry (*N* = 28) are presented using descriptive summary metrics, specifically means, standard deviations (SD), and corresponding 95% confidence intervals (CI). Categorical outcomes, including patient weaning success classifications, are expressed as counts (*n*), percentages (%), and 95% exact confidence intervals to account for small cell sizes.

To evaluate changes in respiratory dynamics within the same patient before and after device utilization, a **paired *t*-test** was executed on patient tidal volumes (mL) to establish the mean difference between baseline positive-pressure MV settings and active DP System support, generating a cohort-specific *t*-value and *p*-value. Primary statistical reporting is heavily centered on a detailed descriptive analysis of the complete cohort (*N* = 28) to reflect real-world clinical parameters transparently. Subgroup analyses comparing the complete 24 h weaning cohort (*n* = 18) against the partial weaning cohort (*n* = 8) are presented using descriptive summary metrics with corresponding 95% confidence intervals (CI). While exploratory independent two-sample *t*-tests were calculated across baseline continuous factors (age, timing, and pacing volumes), these hypothesis tests are severely underpowered due to the small, highly selected sample size; thus, these exploratory *p*-values must not be used to infer comparative clinical effectiveness or generalizable prognostic associations. Evaluation of changes in respiratory dynamics relies strictly on longitudinal, within-patient paired observations—specifically comparing baseline positive-pressure MV parameters directly to active DP system support metrics within the same individual; thus, these *p*-values must be interpreted as strictly exploratory, with primary clinical emphasis placed on descriptive metrics and corresponding 95% confidence intervals (CI).

Regarding missing data management, a complete-case analysis approach was maintained; because this historical chart review yielded full documentation for all primary weaning and baseline selection parameters across the 28 included individuals, no statistical imputation methods were required.

## 3. Results

Between January 2012 and December 2024, 30 patients with cervical SCI were screened for DP at our institution. Two patients were excluded prior to treatment completion (due to transfer to another hospital), leaving a final analytical cohort of 28 patients. The median post-implantation follow-up duration for the final analytical cohort was 60.0 months (IQR: 36.0–84.0 months), spanning a total range of 12.0 to 120.0 months. The final study cohort had a median age at injury of 24.0 years (interquartile range [IQR]: 15.0–33.0 years; range: 5–62 years). The cohort comprised 22 males (78.57%) and 6 females (21.43%). All patients were maintained on invasive MV via tracheostomy prior to DP implantation, with a median pre-implant ventilation duration of 13.0 months (IQR: 10.5–16.0 months).

Out of 28 included patients, 26 were successfully weaned (92.86%; 95% CI: 77.42–98.01%), while 2 patients (7.14%) failed to achieve the minimum criteria for pacing independence. Complete 24 h weaning was achieved by 18 patients (64.29%), and partial weaning was achieved by 8 patients (28.57%). For the cohort achieving partial weaning, nocturnal MV remained necessary.

**Figure 3:**
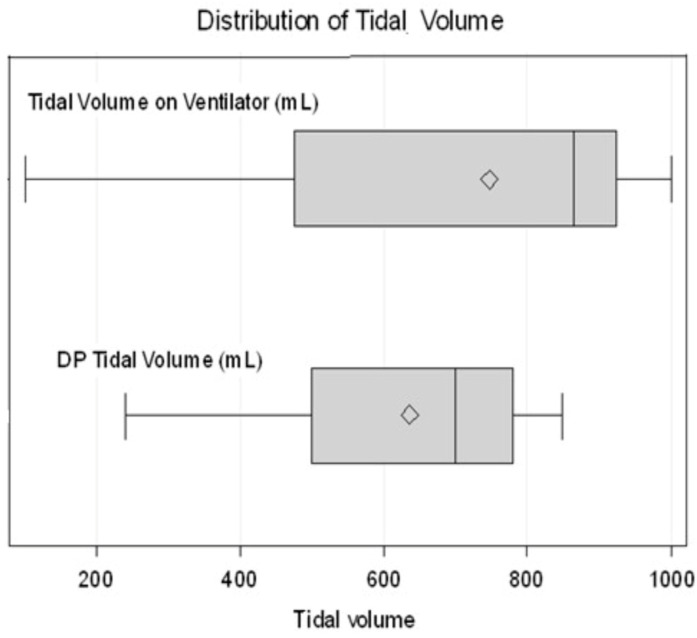
Longitudinal within-patient comparison of respiratory dynamics between positive-pressure MV and active DP support. The open diamond symbols represent the mean values.

Liberation from MV and the use of DP was associated with a reduction in tidal volume. The mean MV tidal volume was 771.8 mL, compared to a mean DP tidal volume of 631.1 mL (mean paired difference of 140.7 mL) (Figure 3).

A subgroup analysis was conducted comparing patients who achieved complete weaning (*n* = 18) to those who achieved partial weaning (*n* = 8). There was no significant difference between the complete and partial weaning groups regarding mean age at injury (20.61 ± 8.56 years vs. 28.25 ± 20.72 years, *p* = 0.1893), mean time from injury to implant (12.78 ± 4.65 months vs. 14.00 ± 5.73 months, *p* = 0.5695), or mean DP tidal volume (663.9 ± 195.7 mL vs. 577.5 ± 240.2 mL, *p* = 0.3418) (Table 1).

Complications and Safety Analysis: Retrospective audit of surgical and follow-up charts revealed zero major intraoperative or postoperative complications, with zero instances of intraoperative deaths, capnothoraces, major hemorrhages, conversions to open surgery, pneumonia, aspiration, or perioperative bleeding events. Over the minimum 1-year follow-up window, the primary documented minor complication was localized exit-site skin irritation observed in 3 patients (10.71%). These mild cases were successfully managed using conservative local care alone, requiring zero oral or intravenous antibiotic administrations. Beyond these minor events, there were zero instances of deep surgical-site infection, exit-site infection, chronic pain, or stimulation intolerance. Assessment of device and wire integrity showed no insulation breaks, electrode dislodgements, or mechanical device failure. Consequently, there were zero reoperations, readmissions, or long-term mortality events across the utilization period. For the two patients who could not be successfully weaned, the therapeutic failure was driven by complex physiological and behavioral barriers rather than device-related structural or mechanical complications. Specifically, both individuals developed severe, intractable hypercapnia (chronic CO_2_ retention) during initial off-ventilator pacing challenges, which was highly indicative of profound, irreversible diaphragmatic muscle atrophy secondary to prolonged pre-implant mechanical ventilation. This physiological limitation was severely compounded by profound patient anxiety and non-cooperation during progressive weaning trials; the acute dyspnea immediately triggered panic, causing tachypnea and recruitment of accessory muscles, which ultimately required the care team to permanently abort the weaning protocol and return the patients to full-time positive-pressure mechanical ventilation for safety. To provide a comprehensive overview of safety outcomes, all tracked intraoperative and long-term postoperative complications are summarized systematically in Table 2 below.

## 4. Discussion

### 4.1. Summary of Key Findings

Out of 28 included patients, an exceptional weaning success rate of 92.86% was achieved, with 64.29% attaining complete 24 h MV liberation and 28.57% achieving partial weaning. The procedure demonstrated an excellent safety profile with zero major intraoperative or postoperative complications, while minor localized exit-site skin irritation occurred in 10.71% of cases.

### 4.2. Comparison with Existing Literature

Our successful weaning rates compare favorably with historical international datasets. Prior landmark multi-center European studies reported that a 4 h ventilator-free window was achieved by 73% and 77% of SCI patients within 6 and 12 months of use, respectively [4]. Similarly, historical data from American clinical trials demonstrated 4 h ventilator-free benchmarks in 88% and 86% of patients [11]. Furthermore, literature tracking complete 24 h liberation rates shows high variability, ranging strictly between 38.1% [4] and 73% [13]. Our observed 24 h complete liberation rate of 64.29% falls within the upper boundaries of these global outcomes.

### 4.3. Interpretation of Discrepancies and Selection Bias

The exceptional 92.86% weaning success rate seen in this cohort must be interpreted with caution and is primarily attributable to several institutional and demographic factors rather than a deviation from global physiological standards. First, severe selection bias is present due to our strict institutional inclusion and exclusion criteria. Patients lacking robust familial or social support networks, those with active psychological disorders, or individuals presenting with severe cardiovascular or metabolic comorbidities that precluded general anesthesia were strictly excluded from receiving the implant. This isolated a highly optimized candidate subpopulation with an inherently greater probability of recovery.

Second, our study population exhibits a distinct youth profile, with a median age of 24.0 years (interquartile range [IQR]: 15–33.0 years). Muscular plasticity, diaphragmatic structural integrity, and general rehabilitative potential are significantly higher in younger individuals compared to older historical cohorts studied abroad, where chronic medical comorbidities frequently compound ventilator dependence. Third, all procedures and postoperative conditioning protocols were executed within a single, highly specialized university tertiary hospital utilizing a dedicated interdisciplinary medical team comprising thoracic surgeons, pulmonologists, and physical rehabilitation therapists. This uniform clinical approach eliminated protocol deviations and optimized pacing conditioning efficacy.

### 4.4. Refinement of Tidal Volume Dynamics

Following the implementation of DP, a reduction in mean tidal volume (paired mean difference of 140.7 mL) was noted compared to baseline positive-pressure MV settings. However, this reduction must be interpreted cautiously and cannot be definitively classified as a direct objective improvement in intrinsic lung function.

Rather than reflecting an optimization of respiratory dynamics, the lower tidal volumes achieved during pacing may indicate that baseline historical ventilator settings during chronic MV were set to inappropriately high target volumes. Alternatively, the lower volumes could represent mild persistent alveolar hypoventilation during early diaphragmatic conditioning. Because longitudinal arterial blood gas parameters and partial pressures of carbon dioxide (PaCO_2_) were not systematically available within our historical dataset to confirm ventilation adequacy, future prospective trials utilizing standardized post-implantation spirometry and blood gas tracking are mandatory to clarify these volumetric changes.

### 4.5. Study Limitations

The primary limitations of this study stem from its retrospective, single-center design and the lack of a concurrent parallel control group managed with standard MV alone. Our small sample size (*n* = 28) limits broader national generalizability and curtails statistical power when conducting subgroup comparisons. Furthermore, because this study relies strictly on historic clinical charts spanning a 12-year window, adjunctive therapy protocols were not consistently documented across all eras. Most notably, objective post-implantation physiological markers—such as longitudinal arterial blood gas measurements, spirometric or pulmonary function tests, and validated multi-dimensional quality-of-life assessment scales—were unavailable. Prospective, multi-center national registries are required to eliminate these historical constraints and validate long-term survival metrics within the region.

## 5. Conclusions

In this selected single-center Saudi cohort of ventilator-dependent cervical SCI patients, laparoscopic DP was feasible and was associated with high rates of partial or complete ventilator-free breathing. Larger prospective multicenter studies with standardized selection criteria, safety reporting, respiratory outcomes, quality-of-life measures, and longer follow-up are needed.

## Figures and Tables

**Table 1:** Duration of DP use and factors impacting weaning duration.

Factors	Category	*n*	Mean (SD)	95% (CI)	*p*-Value
Age at Injury (Years)	24 h	18	20.61 (8.56)	16.36–24.87	
	Partial use	8	28.25 (20.72)	10.93–45.57	
	Difference		−7.64 (13.31)	−19.31–4.03	0.1893
Time from injury to implant (months)	24 h use	18	12.78 (4.65)	10.47–15.09	
	Partial use	8	14.00 (5.73)	9.21–18.79	
	Difference		−1.22 (4.99)	−5.597–3.15	0.5695
Tidal Volume (mL)	24 h use	18	663.9 (195.7)	566.6–761.2	
	Partial use	8	577.5 (240.2)	376.7–778.3	
	Difference		86.39 (209.6)	−97.45–270.2	0.3418

SD—Standard deviation. CI—Confidence interval.

**Table 2:** Structured adverse events and complications tracking within the study cohort (*N* = 28).

Adverse Event	Intraoperative Events (*n*, %)	Postoperative/Long-Term (*n*, %)
Major Complications		
Perioperative Mortality	0 (0.00%)	0 (0.00%)
Capnothorax/Pneumothorax	0 (0.00%)	0 (0.00%)
Major Hemorrhage/Bleeding	0 (0.00%)	0 (0.00%)
Conversion to Open Surgery	0 (0.00%)	0 (0.00%)
Deep Surgical-Site Infection	0 (0.00%)	0 (0.00%)
Pulmonary (Pneumonia/Aspiration)	0 (0.00%)	0 (0.00%)
Minor Complications		
Exit-Site Skin Irritation	0 (0.00%)	3 (10.71%)
Chronic Pacing Pain/Intolerance	0 (0.00%)	0 (0.00%)
Hardware/Mechanical Events		
Electrode Lead Dislodgement	0 (0.00%)	0 (0.00%)
Wire Insulation Break	0 (0.00%)	0 (0.00%)
External Stimulator Failure	0 (0.00%)	0 (0.00%)
Unscheduled Reoperations	0 (0.00%)	0 (0.00%)

## Data Availability

The data that support the findings of this study are not openly available due to privacy and ethical restrictions; however, they are available from the corresponding author upon reasonable request.

## Supplementary Materials

The following supporting information can be downloaded at: https://www.mdpi.com/article/10.3390/medsci14030375/s1, Table S1: The Patient-level baseline characteristics, clinical parameters, and weaning outcomes, Supplementary File S1: Step-by-Step Surgical Technique, Intraoperative Mapping Parameters, and Device Troubleshooting.

## Abbreviations

The following abbreviations are used in this manuscript:

SCI	spinal cord injury
DP	Diaphragmatic pacing
MV	Mechanical ventilation
