# Supplementary material for: Laparoscopic Diaphragmatic Pacing in Spinal Cord Injury Patients with Respiratory Failure: A Saudi Arabian Experience
Source: Med Sci (Basel). 2026 Jul 4;14(3):375. doi: 10.3390/medsci14030375 (PMC13413964; doi:10.3390/medsci14030375)
Supplement: Supplementary file 1 [file medsci-14-00375-s001.zip › Supplementary File S1.pdf]

Port Placement and Patient Positioning

The surgical procedure is performed under general anesthesia with the patient placed in a supine position, and it is mandatory that no neuromuscular blocking agents are administered. Implantation requires the precise insertion of four laparoscopic ports: a periumbilical port dedicated to the laparoscope/optics, two lateral working ports positioned in the right and left upper quadrants to accommodate the diagnostic mapping probe, and one epigastric midline port utilized for the introduction of electrode delivery instruments and the percutaneous exit site from the abdominal cavity for the electrode lead wires. This specific arrangement allows the surgeon to clearly visualize active diaphragmatic contractions, avoid major vessels, and safely route the lead wires out of the abdominal cavity.

Device Model and Electrode Type

The system utilized is the NeuRx Diaphragm Pacing System™ (NeuRx DPS™), which features a four-channel external pulse generator controlled via a temporary clinical station (Figure 1 in main text). It employs PermaLoc intramuscular electrodes anchored directly into the diaphragm muscle tissue using a built-in 5mm blue skirt. An additional indifferent reference electrode is placed subcutaneously in an inferior location to complete the charge-balanced electrical circuit.

Mapping Technique and Stimulation Parameters

The surgeon maps the diaphragm systematically by applying a diagnostic mapping probe directly to various diaphragmatic muscle sites while an Active Sensor Module (pressure transducer) attached via trocar tubing to a lateral trocar measures changes in abdominal pressure. The temporary clinical station interface is configured to default baseline diagnostic stimulation mapping parameters consisting of a pulse amplitude of 25 mA, a pulse width (duration) of 100 μs, a pulse frequency of 20 Hz, a pulse ramp of 0, an inspiration time of 1.1 seconds, and a respiratory rate of 12 breaths per minute (BPM). If severe muscle de-conditioning or lower motor neuron involvement prevents baseline measurements, a diagnostic 'train' pulse—lasting 1.1 seconds in duration—is initiated by depressing the inspiration (INSP) button followed by the stimulation (STIM) button to induce a clear visual contraction.

Motor Point Mapping:

The surgery commenced with a systematic mapping of the diaphragmatic motor points to identify areas of maximal contraction (Figure 2 in main text).

Criteria for “Robust Response” During Stimulability Testing

A robust muscle response is characterized by identifying targeting locations that yield the maximal abdominal pressure change combined with direct visual confirmation of a large portion of the posterior diaphragm contracting. During intraoperative evaluation, these optimal electrode target sites must register a stable A, B, or C connection quality reading on the Clinical Station screen [A superior, B strong, C acceptable]. Any reading displayed as an "X" signifies unacceptable resistance or an open circuit.

Location and Fixation of Electrodes, Exit-Site Management

Electrodes are inserted parallel into the superficial layer of marked primary and secondary motor points, beginning with the posterior sections and anchoring via their 5mm blue skirts. The leads are externalized through the epigastric site and tunneled subcutaneously to a vertical line on the chest or abdomen, with internal wire excess retracted over the liver. Externally, the wires are cleaned, crimped with gold pins, secured into specific slots of an orange connector block, and completely sealed using a silicone-filled strain relief boot.

External Stimulator Programming

Prior to programming, the external pulse generator must operate for a minimum of 1 hour with a test plug installed to ensure its internal storage battery is sufficiently charged.

Clinicians then adjust individual channel settings to match the patient's physiological baseline, ensuring the pulse frequency is capped at a maximum of 20Hz. Target profiles vary significantly by patient type, ranging from default parameters for SCI patients to incremental settings at the threshold of zero pain for ALS patients.

Chronic Pacing Parameter Selection: Following successful implantation, the permanent external pulse generator (EPG) parameters are programmed via the clinical station based on patient sensory baseline types. For spinal cord injury (SCI) patients without sensation, the chronic pacing settings consist of a pulse amplitude of 25 mA, a pulse width of 150 μs, a pulse frequency of 20 Hz, a pulse ramp of 10, an inspiration time of 1.0 to 1.3 seconds (matched to prior ventilator settings or patient comfort), and a respiratory rate of 10 to 14 BPM tailored to meet minute ventilation needs. For SCI patients with retained sensation, chronic parameters are adjusted downward to eliminate discomfort, utilizing a pulse amplitude of 20 mA, a pulse width of 120 μs, a pulse frequency of 18 Hz, a pulse ramp of 10, an inspiration time of 1.1 to 1.3 seconds, and a respiratory rate of 10 to 14 BPM. Chronic pacing stimulation frequency is strictly capped at a maximum of 20 Hz across all patients to prevent diaphragmatic muscle fatigue.

Criteria for Advancing DP Duration

The total duration of off-ventilator pacing periods is progressively increased in a gradual manner over time.

These clinical advancements are based directly on documented improvements in the diaphragm's physical strength.

This methodical progression ultimately enables the patient to spend systematically longer durations safely separated from MV.

Monitoring During Initial Pacing

Clinicians must verify zero cardiac rhythm capture using an intraoperative ECG strip and obtain a postoperative chest X-ray to rule out a capnothorax.

Caregivers must track oxygen saturation via a pulse oximeter, monitor breathing volumes with a Wright Respirometer. A fully operational backup MV system must remain continuously available near the patient during all conditioning sessions.

Management of Device Malfunction

The external stimulator features zero user-serviceable parts and must be returned directly to Synapse Biomedical if a hardware failure or system error message occurs. Technical open-circuit errors ("X" readings) are managed intraoperatively by repositioning the alligator clamps onto the silver pins or using a laparoscopic dissector to manipulate the site. Additionally, routine preventative maintenance requires replacing lithium batteries every 500 hours or standard alkaline batteries every 150 hours to prevent unexpected power depletion
