# Supplementary material for: Laparoscopic Diaphragmatic Pacing in Spinal Cord Injury Patients with Respiratory Failure: A Saudi Arabian Experience
Source: Med Sci (Basel). 2026 Jul 4;14(3):375. doi: 10.3390/medsci14030375 (PMC13413964; doi:10.3390/medsci14030375)
Supplement: Supplementary file 1 [file medsci-14-00375-s001.zip › Table S1.pdf]

Table S1: The Patient-level baseline characteristics, clinical parameters, and weaning outcomes

Patient	Sex	Age at Injury (Y)	Injury Level	ASIA Grade	Time from Injury to Implant (Months)	Baseline Tidal Volume on MV (mL)	DP Tidal Volume (mL)	Current DP Use (Hours/Day)	Weaning Outcome	Nocturnal MV Required?	Complications	Follow-Up Duration (Months)
**1**	Male	22	C2	A	6	800	720	24	Complete	No	None	60.0
**2**	Female	54	C2	A	12	950	600	0	Failure*	Yes	None	24.0
**3**	Male	20	C2	A	18	100	850	24	Complete	No	None	72.0
**4**	Male	28	C2	A	4	800	700	24	Complete	No	None	36.0
**5**	Male	6	C3	B	12	320	250	24	Complete	No	None	108.0
**6**	Female	5	C3	A	24	300	240	8	Partial	Yes	Minor irritation	12.0
**7**	Female	5	C3	A	12	380	300	24	Complete	No	None	84.0
**8**	Female	38	C3	A	8	850	700	6	Partial	Yes	None	48.0
**9**	Male	24	C3	A	16	900	750	24	Complete	No	None	96.0
**10**	Male	58	C3	A	24	1000	650	0	Failure*	Yes	None	30.0
**11**	Male	7	C3	B	12	350	300	14	Partial	Yes	None	54.0
**12**	Male	40	C3	A	14	950	800	24	Complete	No	None	120.0
**13**	Male	26	C3	A	16	1000	850	24	Complete	No	None	60.0
**14**	Male	42	C3	A	6	900	780	6	Partial	Yes	Minor irritation	42.0
**15**	Male	12	C3	A	8	500	400	24	Complete	No	None	78.0
**16**	Male	26	C4	A	13	850	700	24	Complete	No	None	66.0
**17**	Male	62	C4	B	16	900	650	4	Partial	Yes	None	12.0
**18**	Male	20	C4	A	9	800	700	24	Complete	No	None	90.0
**19**	Female	8	C4	A	13	450	350	24	Complete	No	None	54.0
**20**	Female	38	C4	A	17	900	780	6	Partial	Yes	Minor irritation	102.0
**21**	Male	24	C4	A	15	1000	800	24	Complete	No	None	72.0
**22**	Male	22	C4	A	16	980	850	24	Complete	No	None	48.0
**23**	Male	18	C4	A	24	880	750	24	Complete	No	None	114.0
**24**	Male	6	C4	B	12	450	350	10	Partial	Yes	None	36.0
**25**	Male	21	C4	A	13	800	700	24	Complete	No	None	84.0
**26**	Male	24	C4	A	9	900	780	24	Complete	No	None	60.0
**27**	Male	28	C5	B	17	1000	820	14	Partial	Yes	None	120.0
**28**	Male	25	C5	A	12	900	700	24	Complete	No	None	60.0

* Failure reasons were intractable hypercapnia compounded by profound patient anxiety and non-cooperation.
